# Sphingosine Kinase 1 Mediation of Expression of the Anaphylatoxin Receptor C5L2 Dampens the Inflammatory Response to Endotoxin

**DOI:** 10.1371/journal.pone.0030742

**Published:** 2012-02-15

**Authors:** Kurt Bachmaier, Edgar Guzman, Takeshi Kawamura, Xiaopei Gao, Asrar B. Malik

**Affiliations:** Department of Pharmacology, College of Medicine, Center of Lung and Vascular Biology, University of Illinois, Chicago, Illinois, United States of America; The Scripps Research Institute, United States of America

## Abstract

The complement anaphylatoxin C5a has a pathogenetic role in endotoxin-induced lung inflammatory injury by regulating phagocytic cell migration and activation. Endotoxin and C5a activate the enzyme sphingosine kinase (Sphk) 1 to generate the signaling lipid sphingosine-1-phosphate (S1P), a critical regulator of phagocyte function. We assessed the function of Sphk1 and S1P in experimental lung inflammatory injury and determined their roles in anaphylatoxin receptor signaling and on the expression of the two C5a receptors, C5aR (CD88) and C5L2, on phagocytes. We report that Sphk1 gene deficient (*Sphk1^−/−^*) mice had augmented lung inflammatory response to endotoxin compared to wild type mice. Sphk1 was required for C5a-mediated reduction in cytokine and chemokine production by macrophages. Moreover, neutrophils from *Sphk1^−/−^* mice failed to upregulate the anaphylatoxin receptor C5L2 in response to LPS. Exogenous S1P restored C5L2 cell surface expression of *Sphk1^−/−^* mouse neutrophils to wild type levels but had no effect on cell surface expression of the other anaphylatoxin receptor, CD88. These results provide the first genetic evidence of the crucial role of Sphk1 in regulating the balance between expression of CD88 and C5L2 in phagocytes. S1P-mediated up-regulation of C5L2 is a novel therapeutic target for mitigating endotoxin-induced lung inflammatory injury.

## Introduction

Phagocytic cells, macrophages and polymorphonuclear neutrophils (PMNs) from septic patients express inordinate amount of the enzyme sphingosine kinase 1 (Sphk1) compared to macrophages and PMNs from control subjects [1]. Sphk1 phosphorylates sphingosine to form sphingosine-1-phosphate (S1P). S1P in turn signals through heptahelical G-protein-coupled receptors expressed in immune and vascular endothelial cells [2]–[5]. Most cells constitutively express Sphk1, but Sphk1 expression is strongly up-regulated by bacteria and lipopolysaccharide (LPS) [1]. S1P has been reported to reduce neutrophilic inflammation [6]–[10]. In a rat model of acute lung injury, PMN sequestration, production of pro-inflammatory cytokines, NFκB activation, lung capillary leakage, and lung myeloperoxidase (MPO) activity were all reduced by administration of S1P [10].

The physiological concentration in plasma and tissue of S1P is maintained by S1P generation from hematopoietic sources [11], [12]. Genetic deletion of Sphk1 reduces S1P concentrations but is not lethal [13]. However, deletion of both Sphk1 and Sphk2 results in embryos with severe deficiency of S1P generation and lethality in mid-gestation [14]. Tissue concentration of S1P is normally low compared with lymph and blood [5]. Tissue concentrations of S1P are low compared with lymph and blood. This gradient in S1P concentration between blood and tissues contributes to trans-endothelial immune cells trafficking, differentiation, and function [15]–[17]. For example, low concentration of S1P promotes inflammatory cell chemotaxis, whereas high concentration is inhibitory [18], [19] Etiologic agents and mediators of sepsis, including LPS, TNF-α, and complement anaphylatoxin C5a activate Sphk1 in PMNs and macrophages [20]–[23]. The generation of C5a mediates its effects through the heptahelical receptors CD88 (C5aR) and the more recently described C5L2, both of which are expressed on myeloid and non-myeloid cells [24], [25]. In a mouse model of acute lung injury, genetic deletion of C5L2 significantly aggravated the disease [26], and increased lethality in response to LPS challenge [27]. The genetic deletion of CD88 (C5aR) protected mice from acute lung injury [28]. These results suggest opposing roles for the two known C5a receptors in the pathogenesis of lung inflammation, a protective one for C5L2 and a detrimental one for CD88.

Because the mechanisms by which Sphk1 activation and S1P generation reduce neutrophilic inflammation are not well understood, we investigated the possible role of Sphk/S1P signaling axis in regulating the balance between C5L2 and CD88 and how this shift in balance might influence LPS-induced neutrophilic lung inflammation in mice. Our results show that Sphk1 is required to maintain S1P plasma concentration in endotoxemic mice and reveal the essential link between Sphk1 and up-regulation of C5L2. We observed that Sphk1-induced up-regulation of C5L2 is a critical factor preventing endotoxin-induced lung inflammatory injury.

## Results

### Absence of Sphk1 intensifies lung inflammation and increases lethality in endotoxin-induced sepsis in mice

**Figure 1:**
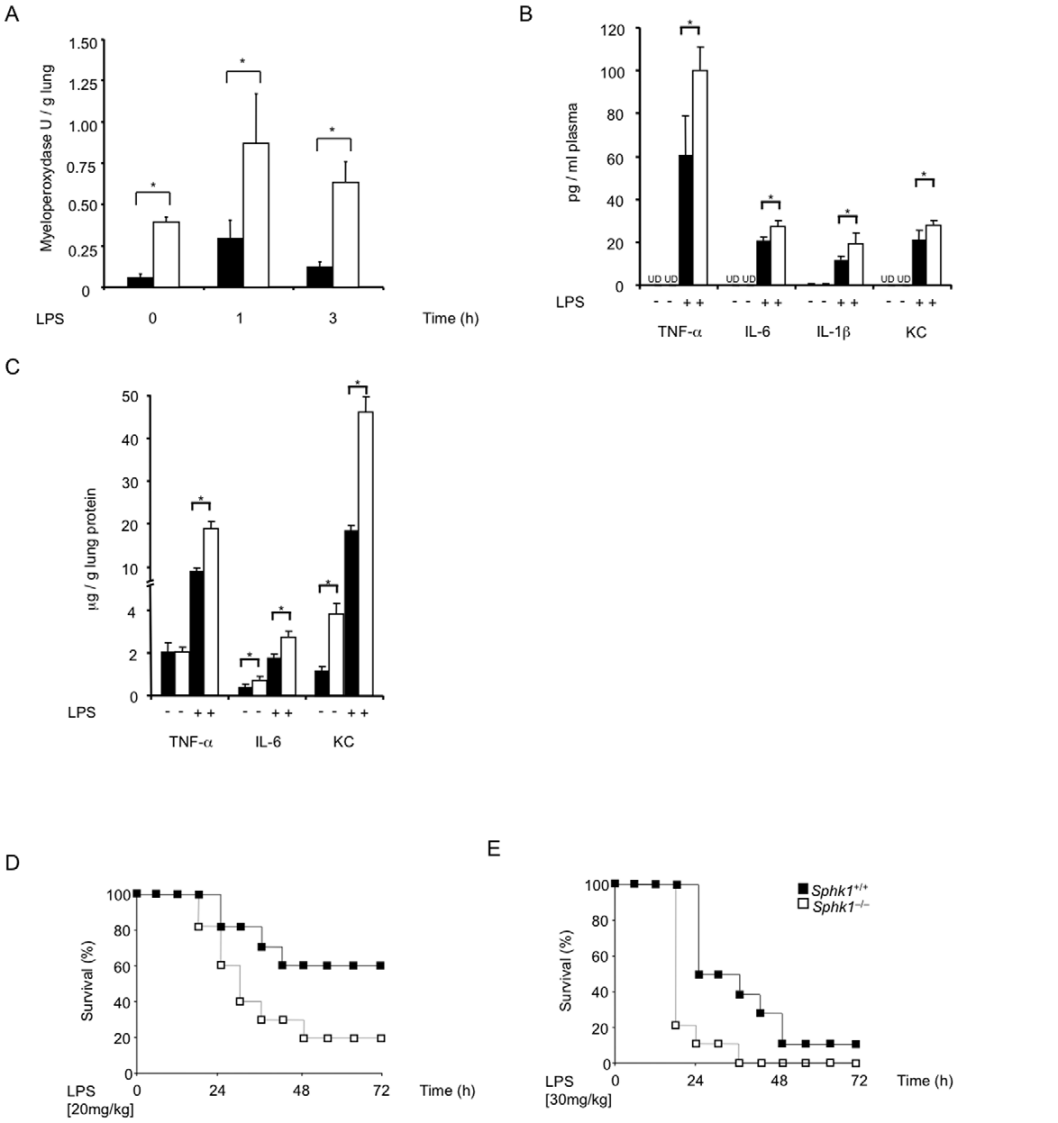
Genetic deletion of Sphk1 amplifies lung inflammation and lethality in mice. (**A**) Lung MPO activity. *Sphk1* ^+/+^ or *Sphk1^−/−^* (n = 10 per time point of each genotype) mice were given LPS i.p. (0.5 mg/kg) and lungs were removed at the indicated times. Error bars represent s.d. *p<0.01 by Student's t-test. (**B, C**) Increased LPS-induced cytokine and chemokine production. TNF-α, IL-6, IL-1β, and KC were measured in plasma (**B**) and TNF-α, IL-6 and KC in lung tissue lysates (**C**) from *Sphk1* ^+/+^ or *Sphk1^−/−^* mice, 1 h after i.p. LPS or saline injection. Error bars represent s.d. *p<0.05 by Student's t-test; n = 5 for each genotype. (**D**) Increased LPS-induced lethality. *Sphk1* ^+/+^ or *Sphk1^−/−^* mice (n = 10/genotype, representative of three independent experiments) were given LPS i.p. (**E**) Increased LPS-induced lethality. *Sphk1* ^+/+^ or *Sphk1^−/−^* mice (n = 10/genotype, representative of three independent experiments) were given LPS i.p. Differences in mortality were assessed by log-rank test (p<0.05). UD, undetected.

Macrophages and neutrophils are hyper-activated in sepsis leading to production of cytokines and chemokines that cause inflammation [29]. LPS activates Sphk1 [21] and Sphk1 protein expression is up-regulated in macrophages and neutrophils from patients with severe sepsis [1]. In mice lacking Sphk1 (Sphk1^−/−^), lung tissue MPO activity, a measure of neutrophilic inflammation, is significantly increased in naïve, non-LPS-challenged mice compared to Sphk1^+/+^ control mice (Fig. 1A). Administration of a sublethal dose of LPS i.p. caused significantly greater MPO activity in Sphk1^−/−^ mice than in Sphk1^+/+^ controls at 1 h and 3 h after LPS injection (Fig. 1A). Sphk1^−/−^ mice have PMNs in blood and bone marrow at numbers and percentages comparable to Sphk1^+/+^ controls (not shown). These data indicate exacerbated inflammatory immune response in the Sphk1^−/−^ mice in response to LPS. Measurements of plasma cytokines tumor necrosis factor (TNF)-α, interleukin (IL)-6, IL-1β and keratinocyte derived chemokine (KC) showed augmented inflammatory response to LPS. At 1 h after i.p. LPS administration, plasma concentrations of TNF-α, IL-6, IL-1β, and KC were significantly greater in Sphk1^−/−^ than in Sphk1^+/+^ mice (Figure 1B). In lung tissue lysate, we found that KC and IL-6 concentrations were basally elevated (Fig. 1C) consistent with the constitutive neutrophilic lung inflammation seen in Sphk1^−/−^ mice (see Fig. 1A). Moreover, after LPS challenge, TNF-α, IL-6, and KC concentrations were significantly greater in lungs from Sphk1^−/−^ mice than from Sphk1^+/+^ littermate controls (Fig. 1C).

As observed previously [30], *Sphk1^−/−^* mice were significantly more sensitive to lethal LPS doses (Fig. 1D). A single dose of 20 mg/kg LPS that was lethal for 40% of *Sphk1^+/+^* mice (LD_40_) was lethal for 80% of *Sphk1^−/−^* mice (Fig. 1D). Others have reported that a wild-type LD_90_ dose of LPS is lethal for only 25% of *Sphk1^−/−^* mice [31]. Thus, we tested the possibility that, when compared to a LD_40_ dose, Sphk1 function is different when a LD_90_ dose of LPS is used. We found that *Sphk1^−/−^* mice showed significantly accelerated lethality to a single dose of 30 mg/kg LPS that is lethal for 90% of *Sphk1^+/+^* controls (Fig. 1E). These data show that Sphk1 is not required for induction of lethality in severe endotoxemia. Sphk1, however, clearly counteracts inflammation and lethality in mice challenged with LPS.

**Figure 2:**
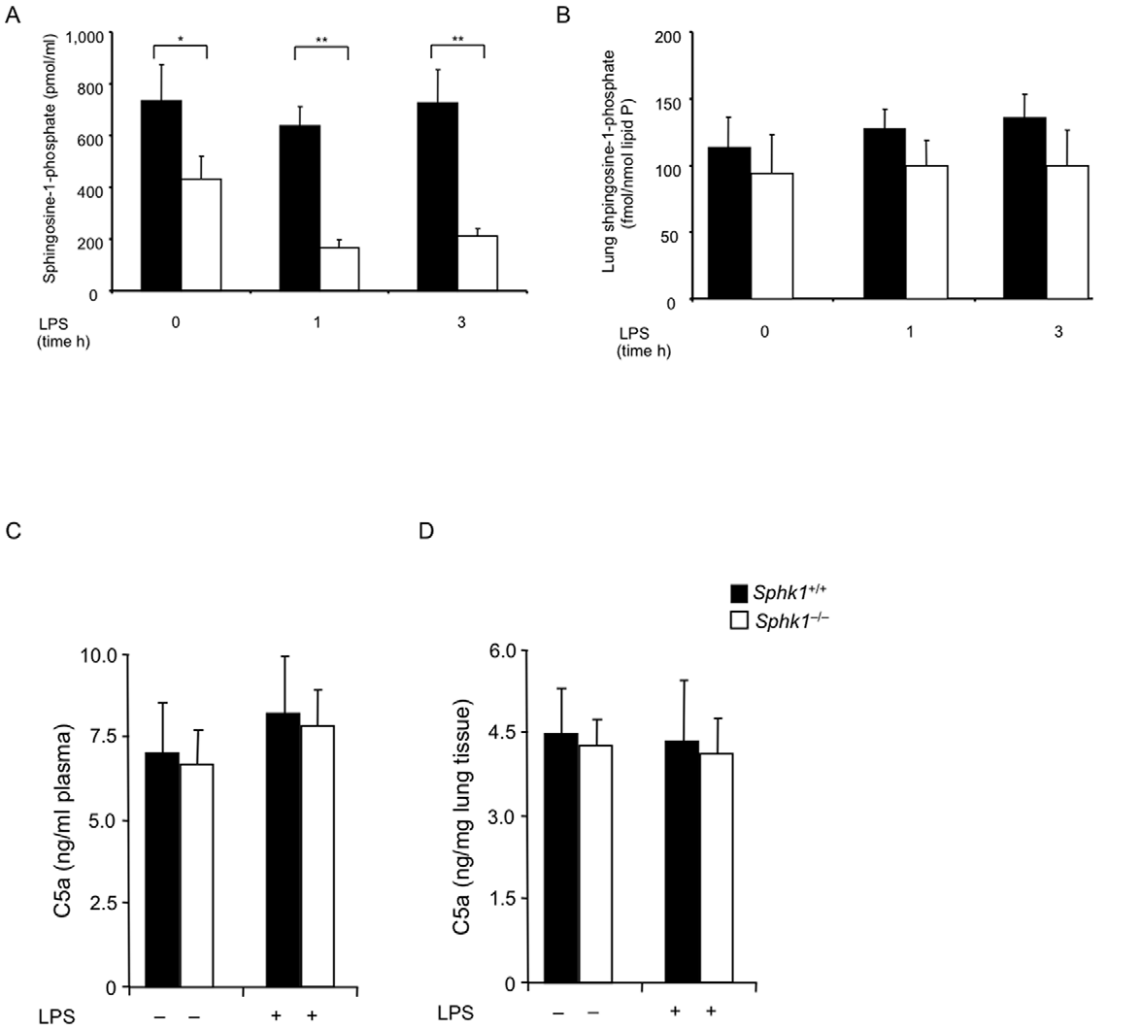
Sphingosine-1-phosphate (S1P) and anaphylatoxin C5a concentrations in plasma and lung tissue. S1P concentrations in plasma (**A**) or lung tissue lysate (**B**) or C5a concentrations in plasma (**C**) or lung tissue lysates (**D**) from *Sphk1* ^+/+^ or *Sphk1^−/−^* mice were determined before or after i.p. LPS challenge (0.5 mg/kg), using the LC-MS/MS (46) or ELISA techniques. Error bars represent s.d. *p<0.005, **p<0.001, by Student's t-test. No significant differences in (**B, C, D**). n = 10 for each genotype and time point.

Pharmacological inhibition of Sphk1 enzymatic activity has been reported as beneficial in mouse sepsis models [1]. Therefore, we measured the product of Sphk1 enzymatic activity, S1P, in plasma and lung tissue. Consistent with an earlier report [13], we found that the plasma S1P concentration was significantly reduced in naïve *Sphk1^−/−^* mice compared to *Sphk1^+/+^* controls (Fig. 2A). After LPS challenge, *Sphk1^+/+^* mice maintained plasma S1P concentrations comparable to non-challenged *Sphk1^+/+^* mice (Fig. 2A). *Sphk1^−/−^* mice, however, failed to maintain their already significantly reduced S1P concentrations. After LPS challenge, S1P concentrations dropped significantly below the plasma concentration of non-challenged *Sphk1^−/−^* littermate controls (Fig. 2A). Lung tissue concentration did not change significantly in either *Sphk1^+/+^* or *Sphk1^−/−^* mice (Fig. 2B). Thus, Sphk1 is required for the S1P gradient between lung plasma and tissue in LPS-induced experimental sepsis. The constitutive low plasma S1P concentration, and the significantly lower S1P concentration in experimental endotoxemia, might explain the PMN lung inflammation and the exacerbated lung inflammatory response to LPS seen in *Sphk1^−/−^* mice.

### C5a-induced reduction in cytokine and chemokine production depends on Sphk1

**Figure 3:**
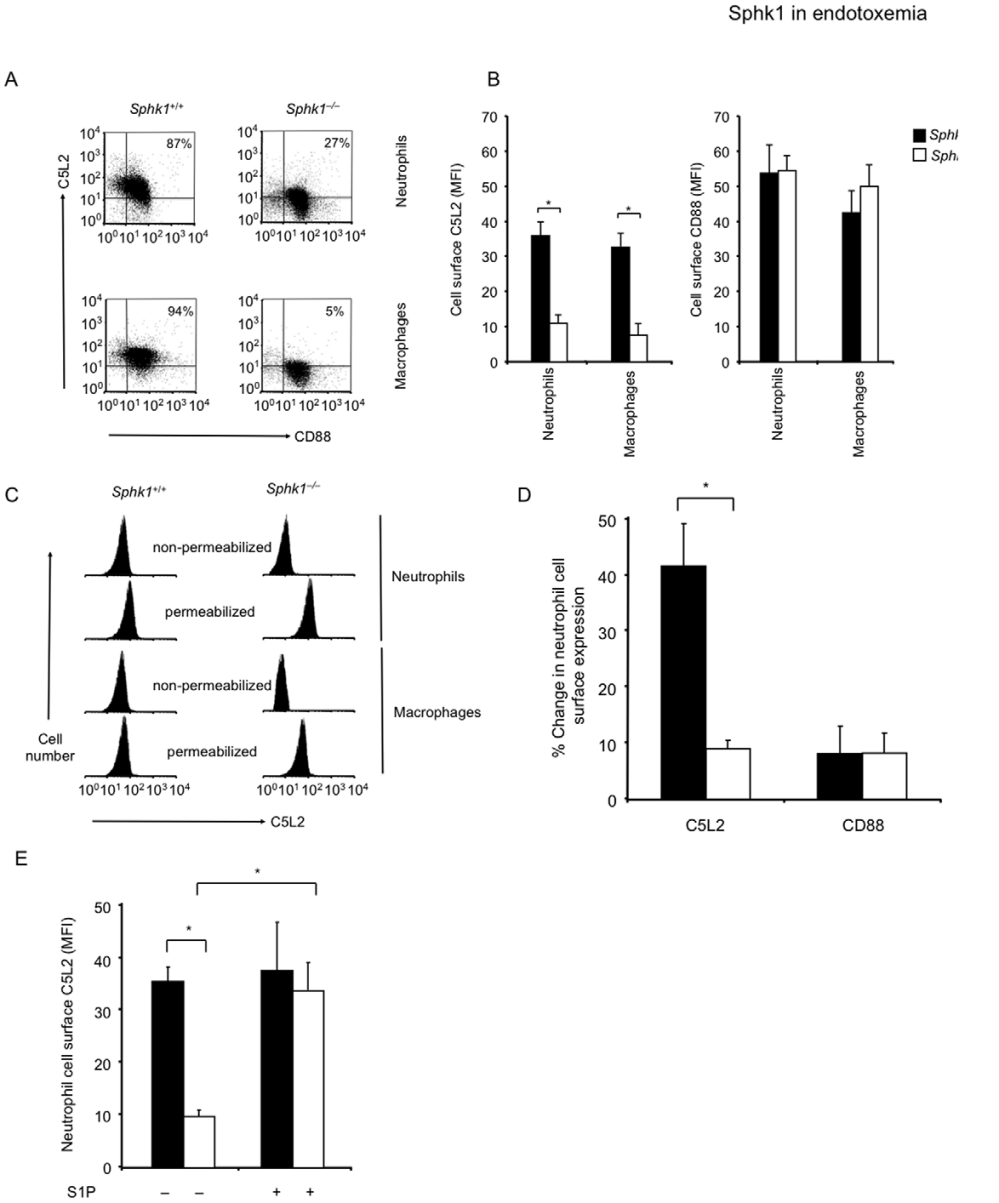
Sphk1 regulates cell surface expression of anaphylatoxin receptor C5L2. (**A**) *in vivo* cell surface expression of anaphylatoxin receptors CD88 and C5L2 in circulating Gr1^+^ neutrophils and F4/80^+^ peritoneal macrophages from *Sphk1* ^+/+^ or *Sphk1^−/−^* mice was determined by flow cytometry. The percentage of C5L2^+^CD88^+^ cells is shown. There is a significant reduction of C5L2^+^ cells in *Sphk1^−/−^* mice. (**B**) Bar graph depicts the mean fluorescence intensity (MFI ± s.d.) of C5l2 or CD88 cell surface expression of cells from five mice per genotype. (**C**) Total C5L2 expression (MFI) assessed by fixing and permeabilizing cells before staining with specific Ab to C5L2. Cell surface expression only, on non-permeabilized cells, is shown for comparison. Representative histograms of C5L2 expression by circulating neutrophils and peritoneal macrophages from *Sphk1* ^+/+^ or *Sphk1^−/−^* mice are shown. (**D**) Percentage change of anaphylatoxin receptors CD88 and C5L2 neutrophil cell surface expression after *in vitro* stimulation with LPS (1 µg/ml) for 1 h compared to non-stimulated controls. (**E**) Exogenous S1P (250 nM) restores C5L2 cell surface expression of *Sphk1^−/−^* PMNs to the level of *Sphk1* ^+/+^ PMNs. Error bars represent s.d. *p<0.05 by Student's t-test. Representative of at least 3 independent experiments with similar results. n.s., not significant.

The complement-derived anaphylatoxin C5a can activate Sphks in phagocytes [20], [23]. C5a is deleterious in sepsis presumably due to its excessive production [32], [33]. We measured plasma and lung tissue C5a concentrations in *Sphk1^+/+^* and *Sphk1^−/−^* mice before and after challenge with LPS. We found no significant increase in C5a concentrations after challenge with LPS (Fig. 2C, D) and genetic deletion of Sphk1 had no effect on C5a concentrations in endotoxemia (Fig. 2C, D). Thus, we surmised that relative expression of the known C5a anaphylatoxin receptors CD88 and C5L2 in inflammatory cells [26], [27], [34] may determine the effects of C5a. It has been shown in mice lacking C5L2 that LPS-induced septic shock is augmented [27], a result comparable to that seen *Sphk1^−/−^* mice (see Fig. 1). Therefore, we analyzed the cell surface expression of the two C5a receptors CD88 and C5L2 in neutrophils from *Sphk1* ^+/+^ or *Sphk1^−/−^* mice. We found that *Sphk1^−/−^* mice had greatly reduced proportions of C5L2^+^ circulating neutrophils and peritoneal macrophages when compared to *Sphk1* ^+/+^ controls (Fig. 3A). PMNs and macrophages from *Sphk1^−/−^* mice had significantly reduced C5L2 cell surface expression compared to *Sphk1* ^+/+^ controls (Fig. 3B). CD88 expression, however, was comparable between genotypes (Fig. 3A, B). Importantly, total C5L2 expression, as assessed by fixing and permeabilizing cells before staining with specific Ab to C5L2, was comparable in neutrophils and macrophages from *Sphk1* ^+/+^ or *Sphk1^−/−^* mice (Fig. 3C) indicating that differences in cell surface expression were not due to differences in total C5L2 protein concentration. PMNs isolated from peripheral blood of *Sphk1* ^+/+^ mice had significantly up-regulated C5L2 cell surface expression in response to LPS, whereas PMNs from *Sphk1^−/−^* mice failed to up-regulate C5L2 (Fig. 3D). CD88 cell surface expression did not change significantly in response to LPS and there was no difference between the genotypes (Fig. 3D). Next to address a potential role of S1P in regulating C5a anaphylatoxin receptor cell surface expression on PMNs, we added S1P to PMNs. S1P restored cell surface expression of C5L2 on PMNs isolated from peripheral blood of *Sphk1^−/−^* mice to the wild type level (Fig. 3E), demonstrating a direct link between S1P and C5L2 cell surface expression.

**Figure 4:**
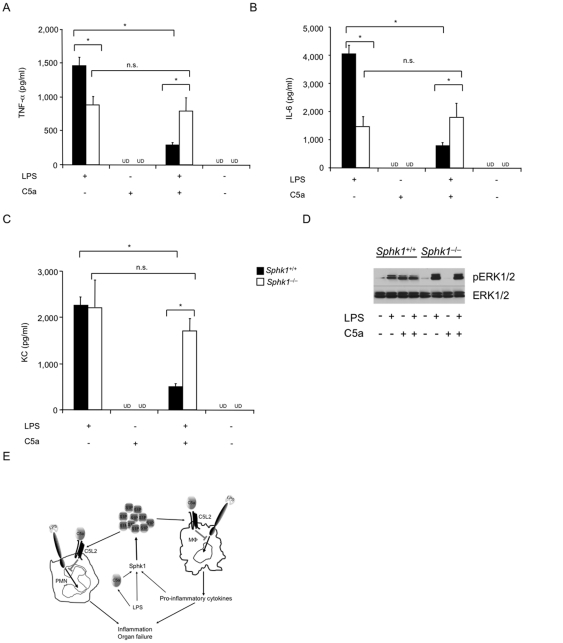
Anaphylatoxin C5a-mediated reduction in cytokine and chemokine production depends on Sphk1. BMDMs from *Sphk1* ^+/+^ or *Sphk1^−/−^* mice were stimulated with LPS (500 ng/ml), with LPS concomitant with C5a (1 nM), or without C5a. Tissue culture supernatants were harvested 2 h (TNF-α) or 8 h (IL-6 and KC) after the addition of stimuli. (**A**) TNF-α; (**B**); IL-6; (**C**) KC. Error bars represent s.d. *p<0.05 by Student's t-test; n = 5 for each genotype; representative for at least three independent experiments. (**D**) Reduced C5a-induced ERK1/2 and activation. BMDMs from *Sphk1* ^+/+^ or *Sphk1^−/−^* mice were stimulated with C5a (10 nM), LPS (500 ng/ml), LPS and C5a, or saline for 5 min. Cell lysates were processed for immunoblotting with indicated antibodies. Representative of 3 independent experiments showing similar results. UD, undetected. (**E**) Model: Reduction of inflammatory cytokine production by phagocytes stimulated with C5a requires Sphk1. The model links Sphk1 activity to the cell surface expression of the anaphylatoxin receptor C5L2. LPS, C5a and inflammatory cytokines activate Sphk1 which is required to maintain S1P during inflammation. S1P regulates C5L2 cell surface expression on phagocytes. C5a, via C5L2 expressed on the cell surface, reduces neutrophil inflammation and inflammatory cytokine production by macrophages.

C5a suppresses TNF-α production by LPS-stimulated human PMNs [35]. To address the effects of C5a on phagocytes lacking Sphk1, we stimulated mouse bone marrow derived macrophages (BMDMs) with LPS in the presence or absence of C5a (Fig. 4A–C). BMDMs from *Sphk1* ^+/+^ mice produced significantly greater TNF-α and IL-6 than BMDMs from *Sphk1^−/−^* mice (Fig. 4A, B), the opposite phenotype from that seen *in vivo* (see Fig. 1B). KC concentrations were similar in both genotypes (Fig. 4C). C5a significantly reduced LPS-induced TNF-α, IL-6, and KC production by BMDMs from *Sphk1^+/+^* mice (Fig. 4A–C). In BMDMs from *Sphk1^−/−^* mice, however, C5a co-stimulation did not reduce TNF-α, IL-6, and KC production in response to LPS (Fig. 4A–C). Stimulation with C5a alone did not induce any detectable cytokine or chemokine production in cells of either genotype (Fig. 4A–C). It should be noted that there was no difference in proliferation or cell death in BMDMs from *Sphk1* ^+/+^ or *Sphk1^−/−^* mice in response to LPS or C5a stimulation (data not shown).

C5a-induced downregulation of cytokine production is mediated in part by extracellular signal-regulated kinase (ERK) [36] and ERK phosphorylation is known to a key downstream event induced by C5a [37], [38]. Moreover, ERK phosphorylation in response to C5a stimulation is dependent on C5L2 expression on macrophages [27]. Thus, we stimulated BMDMs with C5a to assess ERK activation. C5a strongly enhanced ERK1/2 phosphorylation in BMDMs derived from *Sphk1^+/+^* mice (Fig. 4D). By contrast, C5a had little effect on ERK1/2 phosphorylation in BMDMs derived from *Sphk1^−/−^* mice (Fig. 4D). LPS-induced ERK1/2 phosphorylation, and ERK1/2 phosphorylation in response to LPS plus C5a stimulation was normal in BMDMs derived from *Sphk1^−/−^* mice (Fig. 4D). Thus, Sphk1 is required for C5a induced C5L2-dependent activation of ERK1/2. These data link Sphk1 activity to the complement anaphylatoxin C5a and the anaphylatoxin receptor C5L2, and provide a novel mechanism by which S1P counteracts endotoxin-induced inflammation (Fig. 4E).

## Discussion

Here we have identified the critical role of Sphk1 in mediating the expression of the C5a receptor C5L2 in phagocytes and thereby in dampening the inflammatory response to LPS. Sphk1^−/−^ mice have an 80% increase of TNF-α in their plasma, and a 100% increase in the PMN-attracting chemokine KC in their lungs compared to controls in response to LPS. Moreover, the deletion of Sphk1 in mice reduced significantly the lethality in LPS-induced sepsis.

It is not clear whether the function of Sphk1 changes from protective to detrimental when lethality is induced in half of a cohort of mice instead of in 90% of a cohort of mice. Others have reported that a low dose of LPS, which induces lethality in 50% of wild type mice, produced similar lethality in 50% of Sphk1 gene deficient mice. A higher dose of LPS, however, which induces lethality in 90% of wild type mice, caused lethality in only 25% of *Sphk1^−/−^* mice [31]. We found that the presence of Sphk1 was always beneficial for mice challenged with endotoxin. Sphk1 was anti-inflammatory when sublethal doses of LPS are used, and it invariably attenuated and delayed lethality when lethal dose of LPS are used.

The function of the anaphylatoxin receptor C5L2 is still not fully defined [39]. C5L2 plays an important role in various models of sepsis [26], [27], [34], [40]–[42]. Our data suggest that C5a anaphylatoxin signaling in mice is not regulated by the abundance of C5a, because plasma and lung tissue C5a concentration did not change during endotoxin-induced inflammation. We showed that cell surface expression of the anaphylatoxin receptor C5L2 on phagocytes depends critically on Sphk1 in response to LPS challenge. The expression of the anaphylatoxin receptor CD88 in parenchymal cells is up-regulated in experimental sepsis [32], a pathogenetic mechanism that contributes to septic shock and multi-organ failure [34]. Increased C5L2 expression on inflammatory cell surface induced by Sphk1, in contrast, appears to be beneficial in mouse endotoxemia [27]. It is noteworthy that in septic patients, the PMN cell surface expression of C5L2 decreases with the severity of the clinical syndrome [40].

While the complement anaphylatoxin C5 is dispensable for the development of severe sepsis that follows experimental polymicrobial infection [43], the receptors for its activation product, C5a, CD88 and C5L2, are not. In mice, both CD88 and C5L2 contribute to the pathogenesis of polymicrobial sepsis [34]. In contrast, in the more reductionist model used here, endotoxemia, C5L2 signaling is beneficial, via C5a-induced downregulation of cytokine production that is mediated, in part, by ERK activation [36]–[38]. ERK activation in response to C5a, a response that is dependent on C5L2 [27], is absent in BMDMs from Sphk1 mice. ERK activation in response to LPS, however, occurs completely normal in BMDMs from Sphk1 mice.

The phenotype of *Sphk1^−/−^* mice evident in the present study is at odds with the phenotype of wild type mice treated with drugs to inhibit Sphk1 activity or to diminish its expression [1]. There was no defect in NFκB activation in cells from *Sphk1^−/−^* mice [21], [30], [44] whereas mice treated with an Sphk1 inhibitor had a profound defect in NFκB activation [1]. Astonishingly, despite this marked inhibition of NFκB transcriptional activity, there was no defect in clearing polymicrobial infection in mice treated with Sphk1 inhibitor [1]. Adaptive functional redundancy has been suggested to explain the phenotype of *Sphk1^−/−^* mice. For the Sphk1 function uncovered here, the maintenance of S1P plasma levels in endotoxemia, no adaptive compensation appears to have occurred.

It remains unclear how increased expression of the Sphk1 protein or increased Sphk activity in patients' phagocytes correlates with or determines sepsis outcome or lung inflammation. Previous results point to increased Sphk1 protein expression as required to promote inflammation in sepsis or as a compensatory mechanism to curb inflammation. Our results favor the latter protective anti-inflammatory role of Sphk1. Based on our data, we attribute this function of Sphk1 to its essential role inducing C5L2 expression in phagocytic cells and thereby dampening C5a signaling and mitigating the lung inflammatory response to endotoxin.

## Materials and Methods

### Ethics Statement

Mice were bred and maintained according to the guidelines and with the approval of the University of Illinois animal care committee (PHS Animal Welfare Assurance number A3460-01).

### Mice

Mice were bred and maintained under specific pathogen-free conditions at the University of Illinois animal facility. *Sphk1^−/−^* mice, as described [13], were backcrossed into a C57BL/6 background for 9 generations. *Sphk1^+/+^* and *Sphk1^−/−^* mice used for all experiments were maintained in F9. For all experiments, 7–14 wk old mice were used.

### Induction of inflammatory lung injury and lethality

Mice received a single low dose (0.5 mg/kg) of LPS (*E. coli* 0111:B4, InvivoGen) intraperitoneally. For survival studies, mice were injected intraperitoneally with a single dose of LPS (*E. coli* 0111:B4, InvivoGen), 20 mg/kg (LD_40_) or 30 mg/kg (LD_90_), and monitored twice daily for 6 days.

### Myeloperoxidase (MPO) assay

Lungs were perfused with PBS to remove all blood. Lungs were weighed and frozen and stored at −80°C for no more than 1 wk before MPO assay was performed. MPO activity was measured as described [45].

### S1P and C5a measurements

Lungs were perfused with PBS to remove all blood, frozen in liquid nitrogen and processed for LC-MS/MS S1P determination as described [46]. Exogenous S1P (Sigma) (250 nM) was added to freshly isolated PMNs maintained in HBSS buffer containing 0.1% BSA and cells were processed for flow cytometry 1 h after stimulation. For the C5a ELISA, purified rat anti-mouse C5a (BD Pharmingen, 558027) was used as capturing Ab and rat biotinylated anti-mouse C5a (BD Pharmingen, 558028) was used as detection Ab and a standurd curve for C5a protein measurment was established using mouse recombinant C5a (R&D Systems) (see Figure S1).

### Isolation of bone marrow-derived macrophages (BMDM)

BMDM from *Sphk1^+/+^* or *Sphk1^−/−^* mice were obtained and differentiated as described [47]. BMDM were cultured as described [45]. BMDM were placed into 12 well tissue culture plates (1.5×10^6^ cells/well) in IDMD plus 10% heat inactivated FBS. Cells were stimulated with LPS (500 ng/ml) in the presence or absence of mouse recombinant C5a (R&D Systems) (1 nM).

### Cytokine and chemokine concentration measurements

Tissue culture supernatants as well as sera were analyzed for the presence of IL-6, TNF-α, IL-1β, and KC, using the Bio-Plex Multiplex Cytokine Assay (Bio-Rad).

### Phosphorylation and flow cytometry assessment

To determine ERK1/2 phosphorylation, cells were suspended in HBSS buffer containing 0.1% BSA, and stimulated with mouse recombinant C5a (10 mM) at 37°C. Cells were lysed in ice-cold buffer (100 mM Tris-HCL (pH 7.5), 5 mM EDTA, 50 mM NaCl, 5 mM EGTA, 1 mM Na_3_VO_4_, 50 mM NaF, 0.25% Na-deoxycholic acid, 0.1% SDS, 1% Triton ×100, 10 µg/ml protease inhibitors). The antibodies to detect ERK1/2 or pERK1/2 were from Santa Cruz. To determine anaphylatoxin receptor (CD88 and C5L2) cell surface expression, peripheral blood leukocytes, resuspended in HBSS buffer containing 0.1% BSA and stimulated with LPS (1 µg/ml) at 37°C. For flow cytometry, cells were stained in triplicates with anti Gr1 (Pharmingen), anti F4/80 (eBioscience), anti-C5L2 (HyCult biotechnology) or anti-CD88 antibodies (Cedarlane laboratories). To determine the total C5L2 protein expression, cells were permeabilized by using Cytofix/Cytoperm (BD Pharmingen) and stained according to the manufacturers' instructions. Stained cells were analyzed on a LSR flow cytometer (Becton Dickinson). The % of receptor up- or down-regulation was determined as described [45].

## Supporting Information

Figure S1 **Anaphylatoxin C5a-ELISA standard curve.** The standard curve was generated using mouse recombinant C5a as standard and Abs reactive to mouse C5a to capture and detect C5a. Measurements, in triplicate, ± s.d., are shown.
(TIF)
