# Supplementary figures and images for: Sphingosine Kinase 1 Mediation of Expression of the Anaphylatoxin Receptor C5L2 Dampens the Inflammatory Response to Endotoxin
Source: PLoS One. 2012 Feb 15;7(2):e30742. doi: 10.1371/journal.pone.0030742 (PMC3280265; doi:10.1371/journal.pone.0030742)

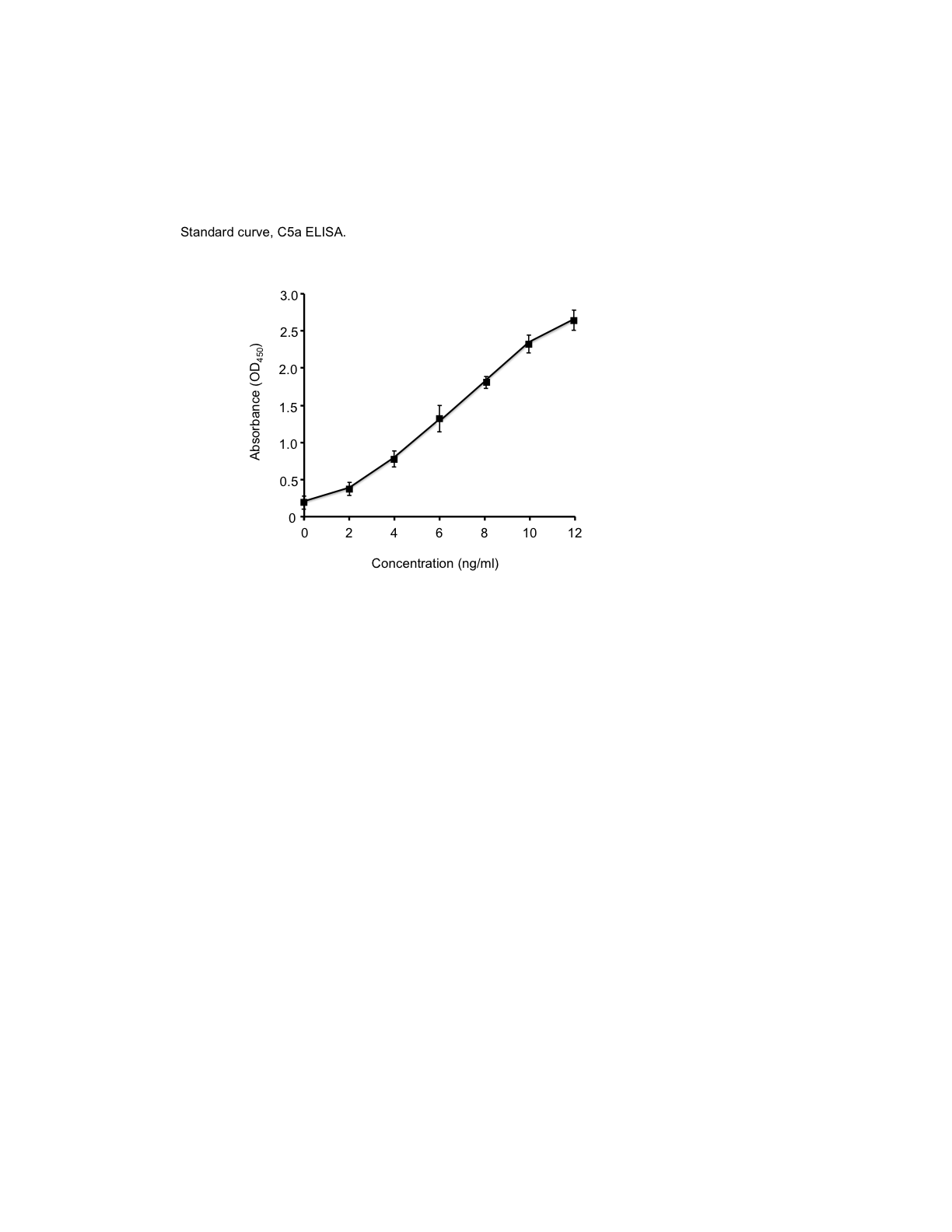

Supplement: Figure S1 — Anaphylatoxin C5a-ELISA standard curve. The standard curve was generated using mouse recombinant C5a as standard and Abs reactive to mouse C5a to capture and detect C5a. Measurements, in triplicate, ± s.d., are shown. (TIF) [file pone.0030742.s001.tif]
